# Valuable interaction with cognitive remediation and optimal antipsychotics for recovery in schizophrenia (VICTORY-S): study protocol for an interventional, open-label, randomized comparison of combined treatment with cognitive remediation and lurasidone or paliperidone

**DOI:** 10.3389/fpsyt.2023.1331356

**Published:** 2024-02-06

**Authors:** Ryotaro Kubota, Satoru Ikezawa, Hideki Oi, Mari S Oba, Shoki Izumi, Ryoko Tsuno, Leona Adachi, Mako Miwa, Shunji Toya, Yohei Nishizato, Daisuke Haga, Tatsuro Iwane, Kazuyuki Nakagome

**Affiliations:** ^1^Department of Forensic Psychiatry, National Center of Neurology and Psychiatry Hospital, Tokyo, Japan; ^2^Department of Psychiatry, National Center of Neurology and Psychiatry Hospital, Tokyo, Japan; ^3^Department of Psychiatry, International University of Health and Welfare Mita Hospital, Tokyo, Japan; ^4^Department of Clinical Data Science, National Center of Neurology and Psychiatry, Tokyo, Japan; ^5^CNS Group, Medical Science, Sumitomo Pharma Co., Ltd., Tokyo, Japan; ^6^One More Job Training Institution, Osaka, Japan; ^7^Kyoto Prefectural Rakunan Hospital, Kyoto, Japan

**Keywords:** antipsychotics, cognitive impairment, cognitive remediation, lurasidone, Neuropsychological Educational Approach to Remediation (NEAR), paliperidone, schizophrenia

## Abstract

**Background:**

Cognitive impairment, a core feature of schizophrenia, is associated with poor outcomes. Pharmacotherapy and psychosocial treatment, when used alone, have inadequate effect sizes for cognitive impairment, leading to recent interest in combination interventions. A previous study examined the additive effect of cognitive remediation on lurasidone in patients with schizophrenia, which was negative. Although improvement in cognitive function was suggested for lurasidone, it was inconclusive because there was no antipsychotic control in the study. To clarify whether lurasidone has a meaningful impact on cognitive function in combination with cognitive remediation, we use paliperidone as a control antipsychotic in this study. We hypothesize that combination with lurasidone will improve cognitive and social function to a greater extent than paliperidone.

**Methods:**

The valuable interaction with cognitive remediation and optimal antipsychotics for recovery in schizophrenia study is a multicenter, interventional, open-label, rater-blind, randomized comparison study, comparing the effect of lurasidone plus cognitive remediation with that of paliperidone plus cognitive remediation in patients with schizophrenia. The Neuropsychological Educational Approach to Remediation (NEAR) is used for cognitive remediation. Eligible patients will be randomized 1:1 to receive lurasidone or paliperidone combined with NEAR (6 weeks antipsychotic alone followed by 24 weeks combination antipsychotic plus NEAR). The primary endpoint is the change from baseline in the tablet-based Brief Assessment of Cognition in Schizophrenia composite T-score at the end of the NEAR combination treatment period. Secondary endpoints will include change from baseline in social function, schizophrenia symptoms, and quality of life at the end of the NEAR combination treatment period. Furthermore, change from baseline to the end of the pharmacotherapy period and change from the end of the pharmacotherapy period to the end of the NEAR combination treatment period will be assessed for all endpoints. Safety will also be evaluated.

**Discussion:**

Achievement of adequate cognitive function is central to supporting social function, which is a key treatment goal for patients with schizophrenia. We think this study will fill in the gaps of the previous study and provide useful information regarding treatment decisions for patients with schizophrenia.

**Clinical trial registration:**

Japan Registry of Clinical Trials ID, jRCTs031200338.

## 1 Introduction

Schizophrenia is a psychiatric disorder presenting with significant impairment in social function, which comprises functions related to a person’s ability to interact with their environment, to live at home and in society, and to maintain communication with others (1, 2). Atypical (second-generation) antipsychotic drugs are effective for positive symptoms of schizophrenia and have comparatively fewer side effects than typical (first-generation) antipsychotics; however, negative symptoms remain in some cases, and they have limited efficacy in improving cognitive impairment (3, 4). Both the European Psychiatric Association and the Japanese society of Neuropsychopharmacology guidelines for schizophrenia primarily recommend atypical antipsychotic drugs (5, 6).

Cognitive impairment is a core feature of schizophrenia (7), which has led to an increased focus on the relationship between schizophrenia and cognitive function in treatment guidelines (5). Among patients with schizophrenia, cognitive impairment is associated with poor social function outcomes and disability, worse community functioning, lower patient quality of life, and increased burden on healthcare services (7–11). Cognitive impairment initially appears around the first schizophrenic episode and remains in the chronic phase, even after the patient reaches remission status (12–14). Cognitive function may deteriorate over time in patients with schizophrenia and can range from near-normal levels to a level of severe deficit (15–17). Up to 75% of patients with schizophrenia experience cognitive impairment (18), which, early in onset, is associated with reduced work-related social function (19). Together, the above information suggests that early intervention is important to improve schizophrenia-associated cognitive impairment.

Interventions available to improve cognitive impairment in patients with schizophrenia include pharmacotherapy and psychosocial treatment. The effect size of atypical antipsychotic drugs on cognitive impairment is reported to be approximately 0.17–0.46 (20), while that of cognitive remediation is around 0.45 (21). Given that the mean deficit in cognitive domains may be 1.0-3.0 standard deviations (SDs) below normal, a comprehensive care program may be important for a sufficient improvement in treatment effect size (22). This has led to recent interest in research aimed at improving cognitive function by combining cognitive remediation and cognition-enhancing drugs to effectively increase treatment effect size (23).

The atypical antipsychotic drug lurasidone is a novel benzisothiazole derivative that exhibits a high binding affinity and antagonistic effect on the dopamine D_2_, serotonin (5-HT)_2A_ and 5-HT_7_ receptors, a partial agonistic effect on the 5-HT_1A_ receptor, and no significant binding to histamine 1 and muscarinic acetylcholine receptors (24). In animal models, lurasidone treatment was associated with neuroprotective effects and better cognitive improvement compared with other antipsychotics (25, 26). Furthermore, results from a clinical study suggest that lurasidone may improve cognitive function in patients with schizophrenia (27). Recent preliminary clinical study findings have demonstrated improved cognitive function with lurasidone in patients with bipolar disorder (28, 29). A previous study has suggested that the combination of cognitive remediation with lurasidone had no greater therapeutic effect on cognitive function than the combination of lurasidone with nonspecific video games (30). Although improvement in cognitive function was suggested for lurasidone, the previous study did not adequately demonstrate whether lurasidone enhances the therapeutic effects of cognitive remediation, given that there was no comparison antipsychotic drug (30).

Herein, we describe the protocol for the valuable interaction with cognitive remediation and optimal antipsychotics for recovery in schizophrenia (VICTORY-S) study, which aims to examine the effects of lurasidone combined with cognitive remediation (the Neuropsychological Educational Approach to Remediation [NEAR]) on cognitive function using the tablet-based Brief Assessment of Cognition in Schizophrenia (BAC App) in patients with schizophrenia (31), by comparing with paliperidone combined with cognitive remediation.

## 2 Materials and methods

### 2.1 Study design

**Figure 1:**
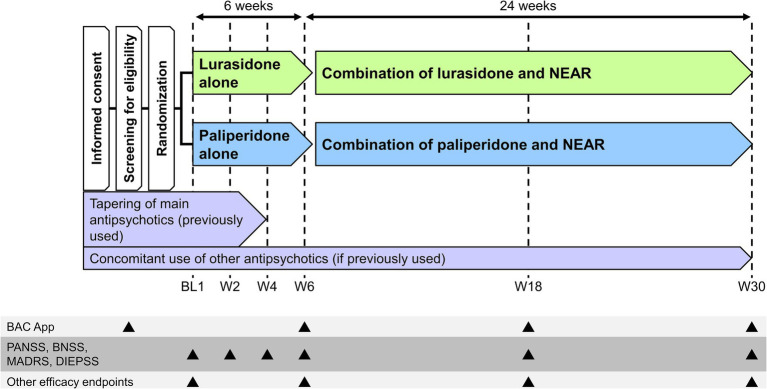
Study design. BAC App, tablet-based Brief Assessment of Cognition in Schizophrenia; BL, baseline; BNSS, Brief Negative Symptom Scale; DIEPSS, Drug-Induced Extrapyramidal Symptoms Scale; MADRS, Montgomery Åsberg Depression Rating Scale; NEAR, Neuropsychological Educational Approach to Remediation; PANSS, Positive and Negative Syndrome Scale; W, Week.

The VICTORY-S study is a multicenter, interventional, open-label, rater-blind, randomized comparison study. The study will be conducted at 17 sites in Japan between 2 February 2021 and 30 September 2025 (Table 1). The study will consist of two periods, a 6-week period of pharmacotherapy alone followed by a 24-week period of pharmacotherapy and NEAR combination treatment (Figure 1). Both patients and therapists will be aware of the group assignment (open-label study design). The allocation for each patient will be disclosed to the therapists, and the endpoint rater will be blinded. Eligible patients will be randomly assigned in a 1:1 ratio to either the lurasidone group (6-week lurasidone alone plus 24-week lurasidone and NEAR combination) or the paliperidone group (6-week paliperidone alone plus 24-week paliperidone and NEAR combination) using the minimization method, with balancing for age (≤39 years and ≥ 40 years), sex, and severity of cognitive impairment measured using the symbol coding task from the tablet-based BAC App (cutoff: 55 points). The cutoff for the BAC App symbol coding was determined by calculating the mean–one standard deviation of the symbol coding scores obtained when the Brief Assessment of Cognition in Schizophrenia Japanese version (BACS-J) was being developed (calculation: 67.4 points [mean] − 12.4 points [SD] = 55 points) (32). An electronic data capture system (HOPE eACReSS) managed by a central data center will be used for randomization.

**Table 1:** List of study sites and principal investigators.

Study site	Principal investigator
National Center of Neurology and Psychiatry	Kazuyuki Nakagome^a^
Hokkaido University Hospital	Naoki Hashimoto
Kohnodai Hospital, National Center for Global Health and Medicine	Toshihiko Ito
Takatsuki Hospital	Yukihiro Nagase
Kanagawa Psychiatric Center	Hisako Taguchi
Kawaguchi Hospital	Taro Takahashi
Inuyama Hospital	Satoru Takazawa
Ainohanazono Hospital	Nobuo Shimizu
Yamaguchi University Hospital	Shin Nakagawa
Kochi Medical School Hospital	Hidetoshi Takahashi
Umibeno-mori Hospital	Kazushi Okada
Amekudai Hospital	Naoki Taira
Fukushima Medical Center, Kokoro no Mori	Yuki Inoue
Hizen Psychiatric Center	Takefumi Ueno
Aoi Clinic	Hiroshi Terada
Tosa Hospital	Yasuhiko Sudo
Shimane Prefectural Psychiatric Medical Center	Hazama Gen-i

^a^Principal investigator of the study.

The study protocol was approved by the Clinical Research Review Board of the National Center of Neurology and Psychiatry, National Research and Development Agency (CRB3200004), and written informed consent will be obtained from patients before enrollment. The study will be conducted in accordance with the principles of the Declaration of Helsinki and the Clinical Trials Act in Japan, and is registered under the identifier jRCTs031200338.^1^

### 2.2 Eligibility criteria

The following inclusion criteria must be met for enrollment in the study: Diagnostic and Statistical Manual of Mental Disorders (5th edition) criteria for schizophrenia; ability to provide in-person written informed consent; outpatient; aged 18–55 years at the time of informed consent; express a preference to switch antipsychotic drug and obtain agreement for this switch by the primary care physician; have presented no risk for self-harm or harming others in the 6 months prior to the date of informed consent; no acute illness requiring treatment; any chronic condition (e.g., hypertension) must be stable with treatment that has been continued for at least 1 month prior to participation; ability to participate in cognitive remediation sessions twice weekly (60–75 min per session); ability to undergo neuropsychological assessment (BAC App); chlorpromazine (CP) equivalent dose(s) of the prior antipsychotic drug(s) not more than 1,000 mg/day in the 30 days prior to the date of informed consent; and no change in the type of main agent of prior antipsychotic medication in the 30 days prior to the date of informed consent.

Exclusion criteria are as follows: a premorbid IQ of less than 70 on the Japanese Adult Reading Test-25 (33, 34); hearing or visual disability; non-native speaker of Japanese; currently receiving lurasidone, paliperidone, or clozapine; currently using three or more antipsychotic drugs; a history of treatment resistance, as evidenced by a failure to respond to at least two antipsychotic drugs when administered for at least 6 weeks at the dose specified on the package insert in the 12 months prior to the date of informed consent; receiving psychotropic drugs known to affect cognitive function, such as methamphetamine; administration of long-acting injections of antipsychotic drugs in the 6 weeks prior to the date of informed consent; a history of electroconvulsive therapy (ECT) in the 6 months prior to the date of informed consent or are expected to require ECT during participation in this study; a likelihood to attempt suicide during participation in this study; a history of intracranial disease or central nervous system disease (e.g., stroke, traumatic brain injury, epilepsy, Parkinson’s disease); a clinically significant abnormality in physical condition; a history of alcohol or drug abuse or addiction in the 6 months prior to the date of informed consent; pregnant or planning to become pregnant; breastfeeding; received cognitive remediation in the 6 months prior to the date of screening; any contraindication to lurasidone or paliperidone; and deemed ineligible for the study in the opinion of the investigator or subinvestigator.

### 2.3 Intervention

During the pharmacotherapy alone period, 40 mg of lurasidone hydrochloride will be administered orally once daily after a meal. The dose will be adjusted as necessary depending on the age and symptoms of the patient; however, the dose should not exceed 80 mg/day. In the paliperidone group, patients will receive 6 mg of oral paliperidone once daily after breakfast. The dose will be adjusted as necessary, not to exceed 12 mg/day and using a daily dose increment of 3 mg with an interval of at least 5 days. In general, tapering of the prior antipsychotic medication (main agent) will be started simultaneously with the start of study drug treatment, initiating the switch from prior medication to study drug. Monitoring of the patient’s condition should be used as a guide for tapering the prior antipsychotic medication (main agent), which should be tapered and discontinued by Week 4. The study drug dose should remain unchanged during the first 4 to 6 weeks following treatment initiation. For drugs included as part of a patient’s prior antipsychotic treatment regimen other than the main agent, continuation of only one drug will be allowed during the study. The main agent is defined as an antipsychotic used at a dose greater than 50% of the daily CP equivalent. The dose should remain the same throughout the study and the drug must have been used for at least 30 days prior to the date of informed consent. The total antipsychotic dose, including the dose of the study drug, should not exceed a CP equivalent dose of 1,000 mg/day. The study drugs will be administered in accordance with the Japanese package insert. The dosage will be adjusted at the discretion of the attending physician within the range specified by the package insert.

Following the pharmacotherapy alone period, the NEAR combination treatment period will begin. NEAR will consist of cognitive task sessions lasting 45–60 min and bridging sessions lasting for 10 to 20 min. These sessions will be conducted twice weekly for 24 weeks. At least one of the practitioners at each site will have received training approved by the developer of NEAR, Alice Medalia (35). To ensure NEAR is being implemented correctly, on-site monitoring will be conducted for some sites using a fidelity scale. Regular supervision meetings will be held by the NEAR practitioners at each facility, either onsite or online. In this period, the dose of the study drug and other concomitant drugs will be maintained without change from the dose used during the pharmacotherapy alone period, but the dose of these drugs can be changed if the attending physician judges it necessary to do so.

### 2.4 Prohibited and restricted concomitant medications and therapies

Prohibited concomitant medications are as follows: as-needed but regular use of antipsychotic medications; adrenaline; strong inhibitors and inducers of cytochrome P450 3A4; and drugs that affect dopaminergic nerve activity, such as psychostimulants and prokinetic agents. Prohibited concomitant therapies are as follows: neuromodulation therapies such as ECT and repetitive transcranial magnetic stimulation therapy, any new psychotherapy other than NEAR, and psychosocial approaches that may affect cognitive function such as daycare and occupational therapy.

Restricted concomitant medications include antipsychotic drugs, psychotropic drugs, and antiparkinsonian drugs. Restrictions for antipsychotic drugs are as follows: antipsychotic drugs for treatment of adverse events (AEs) will be permitted on an as-needed basis up to three times weekly.

Psychotropic drugs are permitted under the following conditions. In general, psychotropic drugs (including antipsychotic drugs other than the main agent) that have been used for at least 30 days prior to the date of informed consent will be continued at the same dose during study participation. If any other psychotropic drug is used on an as-needed basis for treatment of AEs, lorazepam may be used up to 5 times weekly at a dose of ≤1 mg/day. Patients with insomnia may be treated up to 5 times weekly with zolpidem (≤10 mg/dose), eszopiclone (≤2 mg/dose), or zopiclone (≤10 mg/dose). However, none of these drugs should be used within the 12 h prior to cognitive function testing. Antiparkinsonian drugs may be used at an equivalent biperiden hydrochloride dose of ≤3 mg/day. Any concomitant therapy that has been used for at least 30 days prior to the date of informed consent may be continued at the same dosage during participation in the study.

### 2.5 Endpoints

The primary endpoint is the change from baseline in BAC App composite T-score at the end of the NEAR combination treatment period. BACS is a tool to assess both the composite and individual domain scores of cognitive function that are most frequently impaired and most strongly associated with outcomes in schizophrenia (36). The domains assessed include verbal memory, working memory, motor speed, attention, executive functions, and verbal fluency. BACS-J is the validated, Japanese version of BACS (37). The BAC App, which will be used in this study, is a tablet version of BACS/BACS-J (31).

Secondary endpoints include the change from baseline at the end of the NEAR combination treatment period in the Positive and Negative Syndrome Scale (PANSS) (38, 39), Brief Negative Symptom Scale (BNSS) (40, 41), Montgomery Åsberg Depression Rating Scale (MADRS) (42), BAC App subscale T-score, University of California San Diego Performance-based Skills Assessment-Brief (UPSA-B) (43, 44), Specific Levels of Functioning Scale (SLOF) (45, 46), Schizophrenia Quality of Life Scale (47, 48), EQ-5D-5L (49–51), Work Productivity and Activity Impairment Questionnaire (52), and Defeatist Performance Belief (53–55). Change from baseline to the end of the pharmacotherapy alone period and change from the end of the pharmacotherapy alone period to the end of the NEAR combination treatment period in each of these scale scores will also be included as secondary endpoints. Additional endpoints will include the proportion of patients who successfully switch from their prior antipsychotic treatment to the study drugs, the proportion of patients who discontinue the study treatments, the total number of NEAR sessions performed as part of the study treatment, and the proportion of patients who discontinue NEAR. All study raters will have received training in the use of PANSS, BNSS, MADRS, SLOF, UPSA-B, and BACS-J, as well as operational training for the BAC App (VeraSci).

Safety endpoints will include AEs, change in Drug-Induced Extrapyramidal Symptoms Scale (DIEPSS) from baseline and the pharmacotherapy alone period to the NEAR combination treatment period (56, 57), change in DIEPSS from baseline to the pharmacotherapy alone period, vital signs, height, body weight, and laboratory tests.

### 2.6 Sample size

Assuming the standardized group difference of 0.5 based on the effect size (0.82 and 0.32) of previous studies (20, 27, 29, 30), the mixed models for repeated measures (MMRM) requires 64 patients for each group to achieve 80% power to detect a group difference of 0.5 at a two-sided significance level of 0.05 (58). With the anticipation that some patients would fail to switch from their prior antipsychotic drug to the study treatment, a dropout rate of 25% was assumed (27). Based on the above, the target sample size of this study was determined to be 170 patients (85 per group).

### 2.7 Statistical analyses

The efficacy and safety analyses will be based on the full analysis set, which will include all patients who are randomly allocated, undergo treatment, and are evaluated at least once. The per-protocol set will also be analyzed. Two-sided *p*-values will be presented; *p* < 0.05 will be considered statistically significant.

The primary endpoint, change from baseline to end of the NEAR combination treatment period in BAC App composite T-score, will be analyzed using the MMRM to estimate the difference between the groups. Covariates will be allocated group, time point, interaction term of the allocated group and time point, and baseline score. For all other scores of change, the repeated measures will be analyzed using the MMRM, and one-point measures will be analyzed using the linear models.

As a secondary analysis of changes from the end of the pharmacotherapy alone period, MMRM with the inverse probability weighting method will be used to adjust the imbalance between the groups caused by dropouts during the pharmacotherapy alone period. Weights will be estimated using logistic regression with explanatory variables that will be selected from treatment group, patient characteristics, baseline scale scores, occurrence of serious adverse effects, and their interaction terms.

The proportion of patients who complete the switch to the study drug, discontinue the pharmacotherapy alone period, and discontinue the NEAR combination treatment period will be compared between treatment groups using Fisher’s exact test. The number of NEAR sessions completed will be compared between treatment groups using the Wilcoxon test. Subgroup analyses will be performed by baseline BAC App composite T-score, age, sex, duration of disease, and baseline BAC App symbol coding score. Missing data will not be imputed.

AEs will be coded by System Organ Class and Preferred Term per the Medical Dictionary for Regulatory Activities, version J.25.1 or higher. The frequency and proportion of patients reporting AEs will be summarized by time point and group.

All statistical analyses will be conducted using SAS version 9.4 or higher (SAS Institute Inc., Cary, NC, USA) or R version 3.6 or higher.

## 3 Discussion

The VICTORY-S study will examine the effects of treatment with either lurasidone or paliperidone combined with cognitive remediation on cognitive function in patients with schizophrenia. A network meta-analysis of 54 randomized controlled trials that included 5,866 patients with schizophrenia found that lurasidone treatment, when compared with other antipsychotic agents, elicited the greatest improvement in attentional function, working memory, and cognitive composite score (59). Recent efforts aimed at enhancing improvement in cognitive function with treatment have explored combining pharmacological interventions with cognitive remediation; to our knowledge, only one study to date has evaluated the efficacy of combining lurasidone and cognitive remediation for the treatment of schizophrenia (30). In that study, patients received lurasidone combined with either cognitive remediation or video game use. However, cognitive function had recovered by the time cognitive remediation was initiated, and there was no antipsychotic control. For those reasons, the previous study was not able to assess whether lurasidone enhances the therapeutic effects of cognitive remediation. Given the reported beneficial effects of lurasidone on cognitive function (25–29), we hypothesize that the lurasidone plus NEAR combination group may experience a greater improvement in cognitive and social function than the paliperidone combination group. VICTORY-S is the first study to examine the potential of lurasidone to facilitate the therapeutic effects of cognitive remediation for schizophrenia.

Adequate social function supports the ability of patients with schizophrenia to live in social communities. Together with improvement of subjective satisfaction, improvement of social function is considered an important treatment goal for schizophrenia. Given that cognitive function is the factor most related to social function (7), treatment is often aimed at its improvement. Studies have shown that neither pharmacological nor psychosocial treatment alone have adequate effect sizes (0.17–0.46) (20, 21). Though there are few reports of the effectiveness of combination therapy, many clinicians treat their patients with a combination of pharmacological and psychosocial therapy. Cognitive remediation has been shown to improve social function when added to other psychosocial treatments; however, the time and effort needed to maintain combination psychosocial treatments can be a barrier to patient participation (21). Therefore, we plan to examine the efficacy of a combination of cognitive remediation and lurasidone, an approved antipsychotic that has been suggested to improve cognitive function. The results of this study are expected to help guide treatment choices in daily clinical practice. In addition, if combined lurasidone and cognitive remediation therapies elicit stronger improvement in social function than monotherapy, then there is greater hope that patients with schizophrenia will be able to participate in social activities while continuing treatment for cognitive function.

Green et al. proposed a path diagram of (1) cognitive function; (2) defeatist beliefs; (3) negative symptoms; and (4) social function as a mechanism by which cognitive function affects social function (60). With this in mind, we set the change from baseline in BAC App composite T-score as the primary endpoint of this study and set the endpoints in the path diagram as secondary outcomes. This strategy may reveal which parts of the mechanism are affected by pharmacotherapy and which parts are affected by the addition of cognitive remediation. Thus, the results of this study are expected to be comparable with previously published studies. The assumed treatment period for cognitive remediation is 3 to 6 months, based on the average of 16.7 weeks reported in the meta-analysis (21); the present study will evaluate study endpoints at both 18 and 30 weeks, allowing for the evaluation of treatment duration. From this, we hope that the findings of this study will assist clinicians in implementing NEAR more strategically.

Paliperidone was selected as the active comparator for use in this study. Paliperidone, the major active metabolite of risperidone (9-hydroxy-risperidone), has inhibitory effects on D_2_ and 5-HT_2A_ receptors; as such, it is classified as a serotonin–dopamine antagonist (61). Paliperidone is currently approved for the treatment of schizophrenia in various regions, including the United States, the European Union, and Japan, and is considered to be a standard treatment for schizophrenia. In a network meta-analysis of 34 randomized controlled trials of antipsychotic treatment for schizophrenia (62), paliperidone was significantly superior to placebo in all-cause discontinuation rates and ranked highest among the other antipsychotic agents with respect to Surface Under the Cumulative Ranking Curves, indicating the usefulness of this drug in schizophrenia treatment. It has also been shown that paliperidone does not have an adverse effect on cognitive function (63). Based on this information, we chose paliperidone as the active comparator for use in this study.

Based on the results of previous phase 3 clinical trials of lurasidone and paliperidone, the pharmacotherapy alone period is set to 6 weeks in this study. In the previous clinical trials, the PANSS total score significantly improved after 6 weeks of lurasidone or paliperidone treatment compared to placebo (64–67). Furthermore, a previous study of lurasidone reported a significant improvement in cognitive function compared to placebo at 6 weeks (27). The present study will examine not only the effects of the combination of lurasidone and paliperidone with NEAR, but also these monotherapies. Therefore, the efficacy of these study drugs should have reached a steady state prior to the initiation of the NEAR combination treatment, and 6 weeks is considered sufficient as the pharmacotherapy alone period.

Cognitive function in patients with schizophrenia is also thought to be influenced by daily lifestyle, and it has been suggested that aerobic exercise and aerobic exercise combined with cognitive remediation may improve cognitive function (68, 69). It may be worthwhile to discuss the improvement effects on cognitive function between the results of this study (combination of lurasidone with cognitive remediation) and combination of aerobic exercise and cognitive remediation in the future.

## 4 Conclusion

The VICTORY-S study will be the first to examine the potential of lurasidone to enhance the therapeutic effects of cognitive remediation for schizophrenia. The findings from this study are expected to provide useful insight for clinicians who treat patients with schizophrenia.

## Ethics statement

The study protocol was approved by the Clinical Research Review Board of the National Center of Neurology and Psychiatry, National Research and Development Agency (CRB3200004), and written informed consent will be obtained from patients before enrollment. The study will be conducted in accordance with the principles of the Declaration of Helsinki and the Clinical Trials Act in Japan, and is registered under the identifier jRCTs031200338.

## Author contributions

RK: Investigation, Writing – original draft, Writing – review & editing. SaI: Conceptualization, Investigation, Writing – original draft, Writing – review & editing. HO: Conceptualization, Data curation, Formal analysis, Investigation, Validation, Writing – original draft, Writing – review & editing. MO: Conceptualization, Data curation, Formal analysis, Investigation, Validation, Writing – original draft, Writing – review & editing. ShI: Conceptualization, Data curation, Formal analysis, Investigation, Validation, Writing – original draft, Writing – review & editing. RT: Conceptualization, Data curation, Formal analysis, Investigation, Validation, Writing – original draft, Writing – review & editing. LA: Investigation, Writing – original draft, Writing – review & editing. MM: Investigation, Writing – original draft, Writing – review & editing. ST: Conceptualization, Writing – original draft, Writing – review & editing. YN: Conceptualization, Writing – original draft, Writing – review & editing. DH: Conceptualization, Writing – original draft, Writing – review & editing. TI: Conceptualization, Writing – original draft, Writing – review & editing. KN: Conceptualization, Data curation, Formal analysis, Investigation, Validation, Writing – original draft, Writing – review & editing.

## Footnotes

^1^ https://jrct.niph.go.jp/en-latest-detail/jRCTs031200338
